# Lipid Regulated Intramolecular Conformational Dynamics of SNARE-Protein Ykt6

**DOI:** 10.1038/srep30282

**Published:** 2016-08-05

**Authors:** Yawei Dai, Markus Seeger, Jingwei Weng, Song Song, Wenning Wang, Yan-Wen Tan

**Affiliations:** 1 Department of Physics, Fudan University, 200433, Shanghai, P.R. China; 2 State Key Laboratory of Surface Physics, Fudan University, 200433, Shanghai, P.R. China; 3 Institute of Biophysics, Goethe-University Frankfurt Max-von-Laue-Str. 1, 60438, Frankfurt/Main, Germany; 4 Shanghai Key Laboratory of Molecular Catalysis and Innovative Materials, Department of Chemistry and Institutes of Biomedical Sciences, Fudan University, 220 Handan Rd., 200433, Shanghai, P.R. China

## Abstract

Cellular informational and metabolic processes are propagated with specific membrane fusions governed by soluble N-ethylmaleimide sensitive factor attachment protein receptors (SNARE). SNARE protein Ykt6 is highly expressed in brain neurons and plays a critical role in the membrane-trafficking process. Studies suggested that Ykt6 undergoes a conformational change at the interface between its longin domain and the SNARE core. In this work, we study the conformational state distributions and dynamics of rat Ykt6 by means of single-molecule Förster Resonance Energy Transfer (smFRET) and Fluorescence Cross-Correlation Spectroscopy (FCCS). We observed that intramolecular conformational dynamics between longin domain and SNARE core occurred at the timescale ~200 μs. Furthermore, this dynamics can be regulated and even eliminated by the presence of lipid dodecylphoshpocholine (DPC). Our molecular dynamic (MD) simulations have shown that, the SNARE core exhibits a flexible structure while the longin domain retains relatively stable in apo state. Combining single molecule experiments and theoretical MD simulations, we are the first to provide a quantitative dynamics of Ykt6 and explain the functional conformational change from a qualitative point of view.

Membrane fusion is a fundamental biochemical process in all membrane trafficking events. The soluble N-ethylmaleimide sensitive factor attachment protein receptors (SNARE) complex mediates membrane fusion mechanisms, which takes a central place in exocytosis, endocytosis, and neurotransmitter release process in cells^1^,^2^,^3^,^4^,^5^,^6^. Abnormal assembling, disassembling, or membrane fusion of SNARE-core-complexes (SCC) are identified to lead to mental diseases, such as schizophrenia, chronic depression, and suicidal behaviour^7^. Crucial SNARE activities, including vesicle-target protein recognition, association, and dissociation, are governed in part by conformational changes of SNARE proteins. Especially when forming the fusion complex, SNAREs assume a very stable structure yet can be unzipped for recycling to the next trafficking event. Therefore, studying the mechanism and determining the energetics of intramolecular conformational changes of SNAREs may help us to understand the basic mechanism and the working model of SNARE proteins. Most SNARE proteins consist of three domains: a variable N-terminal regulatory domain, a conserved central 60–70 amino acid ‘SNARE core’ that mediates the self-assembly of the four helix-bundle SNARE core complex, and a C-terminal transmembrane domain^8^. For R-SNAREs, such as Ykt6, Sec22B, and VAMP7 in mammals, the N-terminal domain plays a role by interacting with the SNARE core domain and inhibiting its SNARE function^9^,^10^,^11^. The profilin-like N-terminal domain of Ykt6 has been recognized to be part of a larger family of domains with virtually identical folds despite little sequence similarity. These domains have been termed ‘longin’ domains^9^. Unlike most other SNARE proteins, Ykt6 does not contain a transmembrane domain for stable membrane association. Instead, it contains a C-terminal ‘CCAIM’ motif which can be palmitoylated at the first cysteine and farnesylated at the second cysteine to form a lipid membrane anchor^12^,^13^. A striking feature of Ykt6 is that it exists in both membrane-attached and soluble cytosolic pools and undergoes cycling between membranes and cytosol^12^,^13^,^14^,^15^,^16^. The conserved SNARE core domain mediates the assembly of the four-helix bundle SNARE complex and N-terminal longin domain plays an important role in modulating the SNARE function and the structural diversities^17^.

Previous studies claimed a potential model such that single-lipidated (i.e. farnesylation at Cys195) Ykt6 exists in the cytosol and adopts an auto-inhibited conformation via farnesyl-dependent interaction between its SNARE core and longin domain^18^. Crystal structure and Nuclear Magnetic Resonance (NMR) studies on rat Ykt6 discovered that the binding of the lipid dodecylphosphocholine (DPC) leading to the formation of a stable complex^19^. Since the hydrophobic binding site of DPC is close to the farnesylation location, the stable Ykt6/DPC structure (PDB-ID: 3KYQ) was suggested to be the farnesylated form of Ykt6. However, it is difficult to resolve further information on alternative conformations or even the dynamics of rYkt6 protein. The lack of farnesylated Ykt6 structure allows room to speculate the real mechanism of Ykt6 when interacting with lipids. Other studies claimed that longin domains exhibit versatile structures which are proposed to regulate the overall SNARE activities as well as to perform essential roles in transport reactions^8^,^20^,^21^,^22^,^23^,^24^,^25^,^26^. To the best of our knowledge, the conformations of Ykt6 in these *in vivo* enzymolysis studies were mostly inferred from comparison of mutants with wild type proteins^15^,^18^. These methods can neither observe the conformational change nor measure the dynamics directly.

In this work, we aim to conduct quantitative measurements of the conformational dynamics of rYkt6 and provide a benchmark result for theoretical studies of Ykt6 functional principles. For this purpose, single molecule fluoresence methods are powerful means to investigate SNARE systems at molecular level. We explored the conformational distributions and dynamics using single-molecule Förster Resonance Energy Transfer (smFRET)^27^,^28^,^29^,^30^,^31^. Judging from the size of longin domain and the SNARE core of Ykt6, the conformational dynamics of this intramolecular movement may be in the sub-millisecond range. In this case, it would be very challenging for smFRET to acquire the intramolecular conformational dynamics. Therefore, we utilized Fluorescence Cross-Correlation spectroscopy (FCCS) based on Förster Resonance Energy Transfer (FRET) due to its sensitivity to fluctuations on the microsecond time scale.

Here, *in vitro* single-molecule and molecular dynamics (MD) studies are combined to investigate the intramolecular interaction of rYkt6 between longin and SNARE core. To eliminate solubility problems from the ‘CCAIM’ motif, rYkt6ΔC lacking the ‘CCAIM’ motif is our choice of construct. We discover that the dynamics could be regulated by lipid DPC. In the absence of DPC, open and close dynamics between the longin and the SNARE core are readily detected. With increasing DPC, Ykt6 appears to reside mostly in one of the conformational states. It represents a coordinated action of the longin domain, the SNARE core, and the attached lipids. Furthermore, SNARE core contributes to the flexible conformations in the simulations. On the contrary, the Ykt6/DPC results reflect that when Ykt6 is regulated by DPC, it would prefer a stable and rigid state possibly facilitating the formation of a SNARE complex.

## Results

### Single-molecule FRET disclosed two discrete conformations between the longin domain and the SNARE core of Ykt6

**Figure 1:**
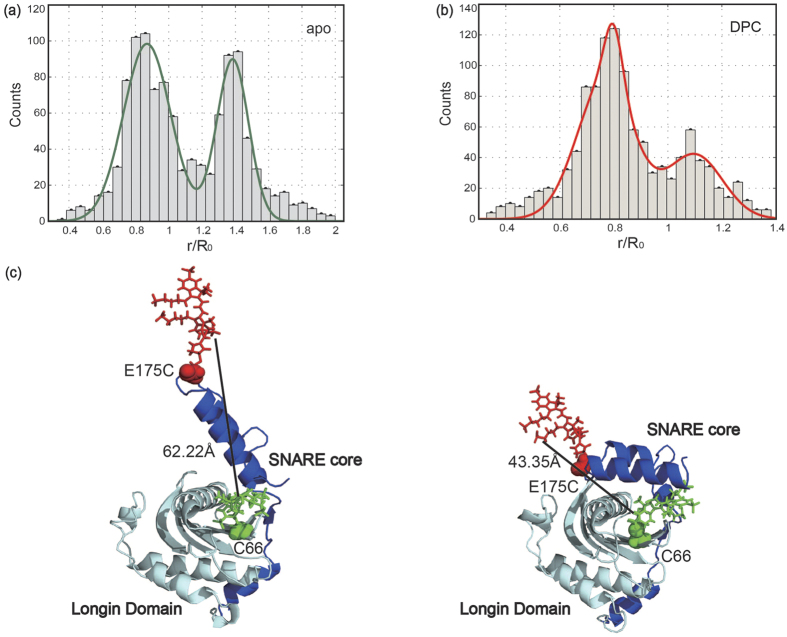
SmFRET Distance Distributions and structures of Ykt6 in the apo and with DPC conditions. (**a**) Apo condition: Distribution of SNARE core to longin domain distances from smFRET results for doubly labeled Ykt6 molecules before adding DPC. Fitting results with two Gaussians are plotted in green (Peaks are centred at 0.85R_0_ and 1.39R_0_, respectively). (**b**) DPC condition: Distributions of distance from smFRET results for double labeled Ykt6 molecules in the presence of DPC environment (Ykt6:DPC = 1:10). The Gaussian fitting lines also are included in red line (Peaks are centre at 0.82R_0_ and 1.09R_0_, respectively). (**c**) Two structures predicted by our MD simulations showing the structures of doubly labeled Ykt6 in open (left) and closed (right) states. Both structures were simulated based on PDB structure 3KYQ, which was closer to the closed form. Structures of dyes were simulated with assumed structures of Alexa 555 (green) and Alexa 647 (red).

Single-molecule FRET assay reports the possible number and distribution of conformational states through measuring the distances by efficiency. We designed a single-point mutation protein construct rYkt6ΔC/E175C for this experiment. The native Cysteine in rYkt6 is utilized as one of the labeling sites. This FRET construct exhibits physicochemical features close to that of wild-type Ykt6ΔC (Fig. 1(c); Supplementary note, Figure S1). Doubly labeled protein samples were immobilized using streptavidin conjugation on the biotinPEG/mPEG-coated glass slide surface. FRET data were acquired via a Total Internal Reflection Fluorescence Microscope (TIRFM) equipped with a dual-view channel splitter and an EMCCD.

Data shown in Fig. 1(a) are the smFRET distances measurement collected from 26 trajectories of rYkt6ΔC/E175C in the absence of DPC. Two distinct FRET distance peaks were yielded. Hence, Ykt6 in its apo condition is present in two different states and might undergo conformational change. When fitted by two Gaussian peaks, these two FRET distributions centred at distance D_1_~0.87R_0_ and D_2_~1.38R_0_ (Fig. 1(a). Here, D_1_ and D_2_ denote the centres of fitted Gaussian peaks with larger and smaller FRET efficiency, respectively. Meanwhile, 28 trajectories of Ykt6 at the presence of DPC (ratio Ykt6:DPC = 1:10) also generates two peaks (Fig. 1(b)). The major conformation distribution was observed at D_3_~0.80R_0_ with a minor distribution peaked at D_4_~1.10R_0_. D_3_ and D_4_ are defined similarly as D_1_ and D_2_.

In apo environment, Ykt6 has one opened state (centred at 1.38R_0_) and one closed state (centred at 0.87R_0_). In contrast, by adding DPC, Ykt6 has mostly resided in a closed state (centred at 0.80R_0_), whereas the population shifts from 55% to 71%. These results indicate a more mobile form of Ykt6 in the apo environment. In the DPC environment, the molecules prefer the closed conformation.

### Dynamics between the longin domain and SNARE core diminishes with increasing DPC

Achieved smFRET distributions had shown two possible conformations between longin domain and SNARE core of Ykt6. In order to investigate the dynamics between the two domains, we measured the temporal information of the conformational changes by FCCS. Moreover, FCCS experiment is compatible with freely diffusing rYkt6ΔC/E175C sample without the need to immobilize the molecules and the fluorescence signals can be detected in samples of ultra-low concentration.

Through comparing the time shift between normalized time dependent auto-correlation and cross-correlation functions of rYkt6ΔC, FCCS experiment could determine the dynamics covering a wide range of timescales from nanoseconds to seconds. However, FCCS relies heavily on model fitting. Therefore, standard and well-studied specimens (Rhodamine 6G and analogously labeled Poly-Proline peptides) were examined independently to determine pre-defined constant variables relating solely to our setup. These pre-defined constant variables include the effective number of molecules N, the focal volume p, the amplitude of triplet component *f* and the triplet constant *τ*_*T*_. Poly-Proline_15_ labeled with Alexa 488 and Alexa 647 were used to determine parameters p, *f*, and *τ*_*T*_. Likewise, we used Rhodamine 6G to get the lateral dimension of the focus μ and the average transit time *τ*_*D*_ through the focus (Figures S3 and S4; Tables S1 and S2). We switched the FRET pair from Alexa 532/647 used in smFRET to Alexa 488/647 because the latter has minimum spectral overlap and therefore reduced crosstalk in the correlation functions.

**Figure 2:**
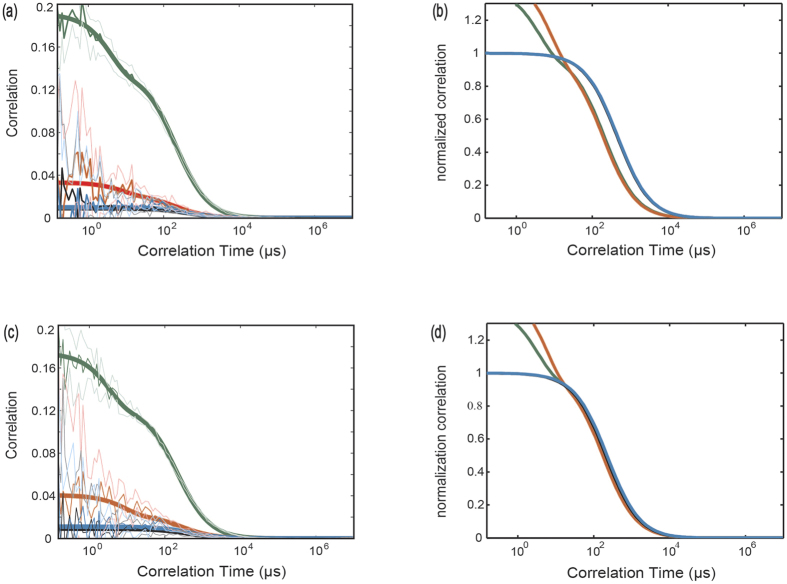
FCCS data for apo and saturated DPC (Ykt6:DPC = 1:2) conditions (green: AC-d; red: AC-a; black: CC-da; blue: CC-ad). (**a**) Raw FCCS time correlation function data of rYkt6ΔC (all FCCS experiments are done on samples labeled with Alexa 488/647 FRET pairs) in the apo environment free of DPC. The thin fluctuated lines are the original data, the bold coloured curves are smoothed by increasing the number of binsize in triplet, whereas the thick lines indicate the fitted curves. (**b**) Normalized FCCS data of rYkt6ΔC in the apo environment free of DPC. (**c**) Raw FCCS data of rYkt6ΔC in the environment of Ykt6:DPC = 1:2. (**d**) Normalized FCCS data of rYkt6ΔC in the environment Ykt6:DPC = 1:2.

FCCS data of labeled Ykt6 was measured at a series of DPC concentration ratios from 1:0, 1:2, to 1:10, to regulate the binding proportion (Fig. 2). The concentration of Ykt6 we use is 2 nM. In order to avoid vesicle formation of DPC (critical micelle concentration of 1.1 mM), the mixing with Ykt6 was done at a concentration of 200 nM and incubated with DPC for at least 5 min.

The normalized time-correlation function data shown in Fig. 2(b,c) indicated that with increasing DPC, the shift along time axis between auto-correlation (AC) and cross-correlation (CC) from either donor or acceptor channels decreases and nearly vanishes at a concentration ratio of Ykt6:DPC at 1:2. A control experiment with a ratio gradient from 1:0 to 1:2 confirms that the 1:2 ratio is at saturation (Figure S5). An obvious shift in time axis can be seen at apo Ykt6 environment, but the shift was gone with increasing DPC. It shows that the domains of Ykt6 SNARE core and longin are locked in a rigid state by DPC, most likely the closed state revealed in the crystal structure. Applying the global fitting model to the data with initial values and boundary conditions earned from the independent fits, we acquired the global fit parameters listed in Tables 1 and 2.

Based on the relative time shift of ~200 μs extracted from the FCCS measurements and the populations of the two states from smFRET, we got the kinetic rate constants of apo Ykt6. The rate constants k_open_ is 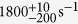, and k_close_ is 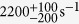. However, when the ratio of Ykt6:DPC equals 1:2, the time shift between AC and CC became negligible (Table 2). This result is consistent with the implications of Wen *et al*.^19^. The results of FCCS and smFRET indicate that the presence of DPC seemed to force Ykt6 to favor a closed conformation, locking down the molecule’s dynamics. Even the minor portion of open state does not exhibit any conformational dynamics at the current timescale.

### DPC restricts rYkt6ΔC into a static state

So far, we have presented smFRET and FCCS data showing the conformational distributions and dynamics of rYkt6ΔC. Our FCCS data suggest that in apo state, there are conformational dynamics between longin and SNARE core of rYkt6ΔC. With increasing DPC, this conformational dynamics diminishes and further disappeared at saturation. However, both apo and DPC smFRET data exhibit two population states, despite a difference in weight. The two peaks observed in the apo experiment can be understood as each molecule would sample both open and closed states during the period of observation. However, we had also observed two peaks in the smFRET DPC experiment, although no dynamics was detected from the FCCS.

**Figure 3:**
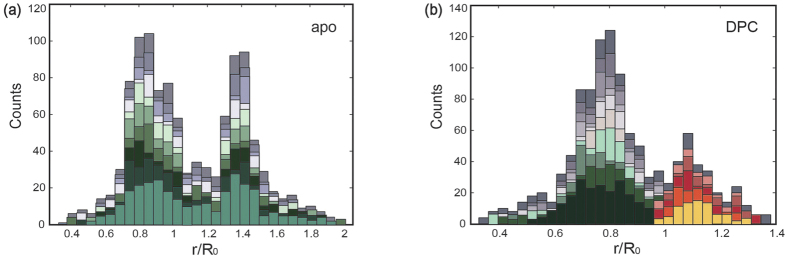
Distance distributions of single trajectory of Ykt6 with/without DPC. (**a**) In the apo condition, we plotted the histogram of Ykt6 SNARE core to longin distances from the smFRET experiment. The whole histogram was divided into different colour blocks. Each colour represents the distance statistics from the same trajectory, and therefore the same molecule. From this plot we can see, each molecule monitored in our apo smFRET experiment contributes to both open and closed states during the observation time. (**b**) When the same plot is generated for the DPC environment (Ykt6:DPC = 1:10), the data from each molecule either contribute to open or closed distance peak. This indicates that the Ykt6 molecule stays in open or closed conformation without exchanging dynamics during the course of observation, which is in the second timescale with a time resolution of 100 ms.

To further investigate the nature of the double populations in smFRET DPC experiment, we extracted every single trajectory and constructed individual efficiency histogram on the same plot in different colours for different molecules. It was found that each trajectory only contributed to either open or closed population (Fig. 3(b)). For reference, we have also constructed the same plot for data acquired in the apo experiment (Fig. 3(a)). In contrary to the apo plot, we have not found any single trajectory such that had contributed to both open and the close peaks from the 28 trajectories processed. Therefore, during the observation period (average trajectory length ~ 8.8 s), none of the Ykt6 molecule undergoes any conformational change under DPC interaction.

Our results show that Ykt6 in apo environment is flexible and transits between the open and closed states freely. Adding DPC molecules forces Ykt6 molecules into different conformations manifested as the two peaks in the smFRET experiment. This observation suggests that the two peaks are resulted from two different forms of DPC binding or interaction. Therefore, there might be more than one DPC binding site on Ykt6 or DPC can interact with Ykt6 in more than one manner.

### Structural diversities behind apo Ykt6 displayed by MD simulations

**Figure 4:**
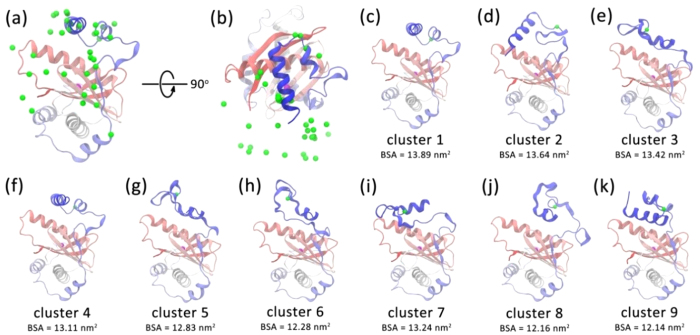
Representative structures with shorter Cys66-Glu175 Cα distances from the MD simulation. Front view (**a**) and top view (**b**) of the cluster centre structures of the snapshots with the Cys66-Glu175 distance ranging from 1.7 to 2.4 nm in the metadynamics simulations using a cutoff of 0.6 nm. The protein is represented by the cartoon mode and is coloured from red to blue. The Cα atoms of Glu175 in the cluster centre structures are represented as green balls and the Cα atom of Cys66 is represented as magenta ball. (**c**–**k**) the centre structures with the BSA between longin domain and SNARE core of more than 12 nm^2^.

With measurements of the dynamics between the longin domain and the SNARE core, we try to decipher the molecular details of apo Ykt6 using MD simulations. According to the FCCS results, the time scale of the conformational changes is around 200 μs, which is beyond the sampling capacity of conventional MD method. Therefore, we used the enhanced sampling method metadynamics to explore the conformational space behind the states along a reaction coordinate, chosen as the distance between the Cα atoms of Cys66 and Glu175 matching the labeling sites in smFRET experiments (see Methods). Three parallel 1.5 μs trajectories with different initial velocities were produced. Since convergence has not been achieved due to the large conformational space, it is difficult to compare the distance distribution based on MD simulations and the smFRET results directly. However, these simulation trajectories could provide us an informative view of the conformational diversity of apo Ykt6. The simulation snapshots were clustered using quality threshold algorithm^32^. The clustering analysis simplifies data interpretation as we can focus on the cluster centre structures. We analyzed the structural properties in separate distances ranges along the reaction coordinate. Illustrated in Fig. 4 are the snapshots with shorter distances, ranging from 1.7 to 2.4 nm, which fall within the ‘closed’ conformation peak observed in the smFRET experiment. Please refer to supplementary information for further analysis on other distances ranges.

For the snapshots with shorter distance range from 1.7 to 2.4 nm, the conformations were divided into 34 clusters with a cutoff of 0.6 nm. The cutoff value was selected so that the representative cluster centre structures are retained (Figure S6). The longin domain of Ykt6 remains well, but the SNARE core takes up variable structures as revealed by the 34 centre structures. The structural diversity can be well described by the positions of the Cα atom of Glu175 (the green balls in Fig. 4(a,b)). In the simulations, Cα of Glu175 was observed to deviate significantly from the position in the crystal structure, moving in a large range around the longin domain. Since the simulations have not achieved convergence, the accurate populations of these clusters were not determined. Nevertheless, we ranked the clusters by the buried surface area (BSA) between the longin domain and the SNARE core. The states with larger BSA are presumably more stable. The 1st and 4th clusters closely resemble the crystal structure (Fig. 4(c,f)). The αF-αG helices pack against the hydrophobic surface on the longin domain and the βF strand retains the backbone hydrogen bonding interactions with the βC strand. In the 2nd cluster, the αF helix is totally uncoiled and forms a β-turn (Fig. 4(d)), indicating that the secondary structures of SNARE core is flexible. The 3rd and 5–8th clusters share a common feature that the αG helix lies parallel with the αA helix. The packing between αG and αA includes interlaced hydrophobic and hydrophilic residues. Secondary structure changes were also observed. For example, the αE and αF helices almost totally unfold and adopt very different conformations. The 9th cluster shows a novel orientation of the αG helix as it lies antiparallel with the αA helix, whereas the αF helix is partially uncoiled (Fig. 4(k)).

Overall, we observed remarkable structural diversity in the distance range corresponding to the apo smFRET experiment. It is demonstrated that the structural variability exclusively originated from the SNARE core, while the longin domain remains its structural integrity. The SNARE core showed structural flexibilities in packing topology as well as secondary structures, which might induce the conformational change of Ykt6.

## Discussion

Among various SNAREs, Ykt6 is exceptional owing to its presence in both cytoplasm and membrane. The conformation of Ykt6 was believed to be linked with whether Ykt6 is anchored on the membranes and or dissolved in the cytoplasm^13^,^16^,^33^. However, the conformations and dynamics of Ykt6 are not clear. Therefore, we focused on exploring the conformations of these two essential functional domains to provide a quantitative description of the dynamics.

The lipid-mediated intramolecular interaction of the longin domain with the SNARE core is observed through smFRET (Fig. 1) and FCCS experiments (Fig. 2). For apo Ykt6, the corresponding time resolved dynamics reveals a conformational exchange in the range of 200 μs. With increasing DPC, rYkt6ΔC lost its flexible properties and stayed in a stable conformation. Consistent with this, we observed that Ykt6 in apo system was very flexible throughout the MD simulations (Fig. 4). Ykt6 exhibits significant structure diversity exclusively originated from the SNARE core in packing topology as well as secondary structures, while the longin domain remains its structural integrity. Since the SNARE core acts as a functional domain which connects longin domain and CCAIM motif, the flexible property is beneficial to control each domain of Ykt6 on forming different conformations in the whole cycling process. In other words, conformational change of rYkt6ΔC in apo state seems to be modulated mainly by the SNARE core.

Recent *in vivo* studies have suggested that there exist different conformations of Ykt6 separately in cytosol and on membrane after palmitoylating^18^,^19^,^33^. Furthermore, the DPC/rYkt6ΔC structure has been suggested to mimic the farnesylated Ykt6 in cytosol^19^. However, the real structure of farnesylated Ykt6 is yet to be acquired. On the other hand, DPC as a lipid, is similar to the material of membrane. Without a confirmed farnesylated structure of Ykt6, it is also reasonable to consider saturated DPC condition as the environment of membrane. Söllner *et al*. hypothesized that SNARE proteins approaching the membrane starts in a closed state, goes through a partly-zippered state and eventually opens to form the complex^34^. In this perspective, DPC experiments can also reflect the condition close to the membrane.

**Figure 5:**
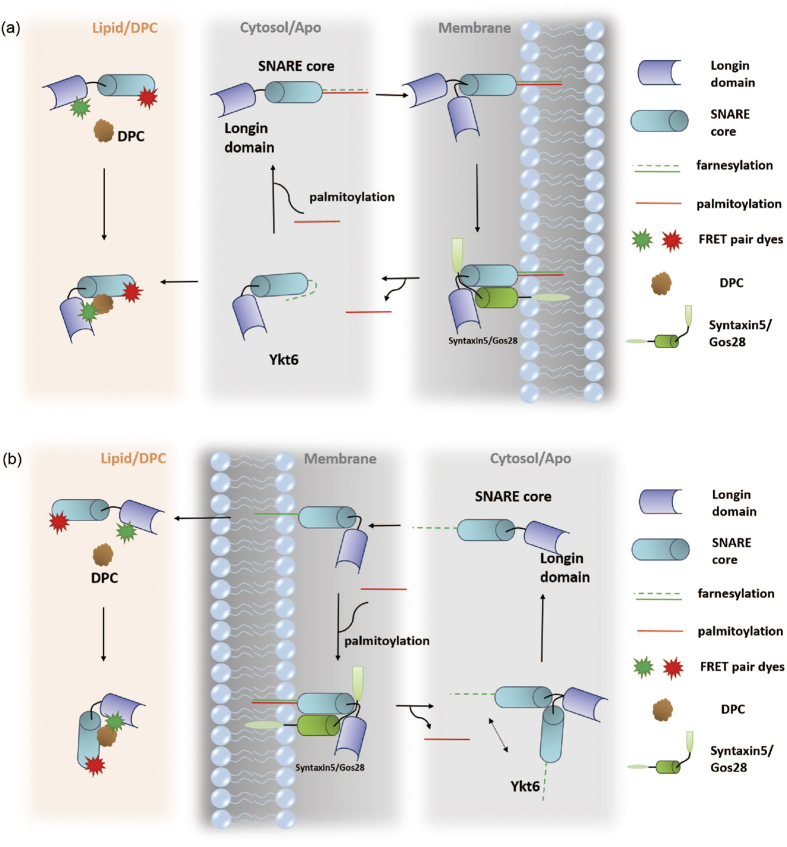
Schematic picture for two hypothesis models depicting that DPC/Ykt6 interaction mimics Ykt6 and membrane interaction *in vivo*. (**a**) Model one: DPC bound is treated as farnesylation state of Ykt6. The farnesylated Ykt6 takes a closed conformation in the cytosol (right panel). There is no longin versus SNARE core conformational dynamics as seen in our DPC FCCS experiment. After being palmitoylated (middle panel), Ykt6 starts to accumulate around the membrane. When Ykt6 is inserted into the membrane after palmitoylation, only longin domain and SNARE core are left in cytosol. After membrane insertion, the longin domain and the SNARE core endure a conformational exchange as seen in the apo smFRET and FCCS experiments. These variable conformations promote Ykt6 to seek for the best formation when forming zipped complexes with Syntaxin5/Gos28 (left panel). (**b**) Model two: DPC as a lipid is treated as the material of the membrane. Our findings suggest that Ykt6 in the cytosol has two different conformations which is similar to the apo condition in our experiments (right panel). These variable conformations can facilitate farnesylation and palmitoylation of CCAIM motif. When Ykt6 is close to the membrane, lipid will help it to form a closed conformation as seen in our DPC experiments. After palmitoylation and therefore membrane insertion, Ykt6 will be kept in a closed and stable state (middle panel). *In vitro* DPC experiment shows that DPC molecule forces Ykt6 into a stable closed conformation (left panel).

Combing our data and previously results, we proposed two different hypotheses for rYkt6 in the cycling process between the membrane and the cytosol. They are illustrated in (Fig. 5a,b). Model one shown in Fig. 5(a) is based on the proposal such that DPC/Ykt6 structure reflects the farnesylation state. This model consists of the following elements: (i) DPC molecules force most Ykt6 into the closed form, which simulates the farnesylation state. Afterwards, the CCAIM motif will be palmitoylated and ready for membrane insertion. When Ykt6 is inserted into the membrane, longin domain and SNARE core are still left in the cytosol. (ii) After membrane insertion, the state of Ykt6 is similar to the apo state such that the molecule assumes two different conformations. (iii) There is a conformational exchange between the two states. In this scenario, our DPC experiment simulates the situation where Ykt6 is farnesylated in the cytosol. This compact and static closed conformation helps the molecule to be better dissolved since most of the hydrophobic residues are buried inside. After palmitoylation, Ykt6 molecules accumulate and insert into the membrane^12^. Anchored onto the membrane, longin domain and SNARE core stay in the cytosol and retain flexible. These variable conformations promote Ykt6 to seek for the best formation when forming zipped complexes from the energetics perspective.

Since there is no farnesylated Ykt6 structure available, we have reservation in treating the DPC interaction as farnesylated Ykt6. Therefore, an alternative model is proposed and outlined in Fig. 5(b). Lipid DPC is treated as the membrane. Our findings suggest that the cytosolic Ykt6s engage as an inactive “ready-to-go” pool of molecules. The second model consists of the following elements: (i) Apo Ykt6 has two different conformations. These variable conformations before membrane insertion can facilitate the palmitoylation of CCAIM motif. (ii) When Ykt6 is close to the membrane, lipid will assist it to form a closed conformation. DPC lipid forces Ykt6 to favor the closed partly-zippered conformation and lowers the conformational flexibilities. (iii) After membrane insertion, Ykt6 will be kept in a closed and stable state.

In summary, the lipid modulated intramolecular conformational distribution and time-dependent dynamics of SNARE protein Ykt6 were measured by smFRET and FCCS. Here, we try to illuminate the working process of Ykt6 from the perspective of conformational dynamics at molecular level. We observe two conformational states of Ykt6 in apo system and provide a quantitative measurement of the dynamics for the first time. From the apo state FCCS measurements, we extracted a conformational dynamics which depicts a mobile and flexible state. Simulated results of rYkt6ΔC conclude that SNARE core has a variable structure and this might be contributing to the dynamic conformations in the apo state. The flexible conformations are beneficial for Ykt6 to be farnesylated and palmitoylated. Under the interaction of DPC, the dynamics is diminishing and the proteins are maintained in a static state. Although whether DPC/Ykt6 can stand for the farnesylation form of Ykt6 is still unclear, we present two hypotheses to foster further discussion and experimental investigations.

## Material and Methods

### Sample preparation

We use Ykt6 from *Rattus norvegicus* as our system. DNA sequences encoding rYkt6 longin domain (residues 1–137), rYkt6ΔC (residues 1–193), and the full-length rYkt6 (residues 1–198) were individually cloned into a modified version of pET32a vector. The double cysteine rYkt6/E175C construct was then expressed, purified, fluorescently labeled, and separated following methods described here. Single point mutation E175C of rYkt6 was carried out using the standard PCR-based mutagenesis method and was confirmed by DNA sequencing. Recombinant proteins were expressed in *Escherichia coli* BL21 (DE3) (TIANGEN BIOTECH CO., LTD) host cells at 16 °C. His_6_-tagged rYkt6 proteins expressed in bacterial cells were purified by Ni^2+^-NTA agarose affinity chromatography followed by anion exchange chromatography. The purified rYkt6-E175C was labeled with maleimide derivatives of Alexa Fluor 488 (AF 488, Life Technologies) and Alexa Fluor 647 (AF 647, Life Technologies) for FCCS experiment. For smFRET experiment, we used Alexa Fluor 555 (AF 555, Life Technologies) and Alexa Fluor 647 as the FRET pair (Förster radius = 51 Å). Free dyes and labeled proteins were separated by a Superdex 75 size exclusion column (GE Healthcare Life Science) equilibrated with the storage buffer (100 mM Tris pH = 8.5; 100 nM NaCl). Further labeling purification was achieved by using sulfo-link (Invitrogen) to remove free thiol-groups which were not bound with dyes. All the purify process used by fast protein liquid chromatography (GE Healthcare Life Science ÄKTA purifier 900). The collected fractions were dialyzed from the FPLC solvents into the storage buffer. The standard sample poly-proline peptide with the sequence Cys-(Pro)_15_-Cys (GL Biochem Ltd) was labeled with AF 488 and AF 647 following the analogous process described previously.

### Setup for smFRET efficiency distribution measurement

SmFRET efficiency distribution measurements were collected by a home-built multi-colour TIRFM built according to Friedman *et al*.^35^ Here we used a Semiconductor 532 nm laser (Coherent, compass 315M-100) as excitation beam which was reflected into a 60x oil-immersion objective (Olympus PlanApo 60x; Cargille Type DF Immersion Oil) by a tiny mirror (Edmund, 4 mm Diameter 45° Rod lens Aluminum Coated, #54-092) and illuminated the sample immobilized on the surface of the slide. The reflected laser excitation was directed out of the optical path by a symmetrical small mirror sitting at 45°. The fluorescence emission from two different dyes was collected though the same objective and passed through a dual-view channel splitter (Photometrics, DV2, filter set: 649/LP as a dichroic, Semrock FF649-Di01-25 × 36; BP580/60M for Donor, Semrock; BP705/100 for Acceptor, Chroma) to display two separated images of emission on one Electron-Multiplying Charge-coupled Device (EMCCD, Andor, DU897E).

### Single-molecule FRET distribution analysis

Single-molecule FRET measurements were carried out on immobilized double-labelled Ykt6 on the biotinylate-PEG-coated slide surface for observation^36^. All experiments were performed at room temperature (~25 °C) in both apo and DPC condition which mimic the cytosol and membrane environment, respectively.

FRET efficiencies were determined in 100 ms bins. The FRET efficiency, E, for each time bin is given by


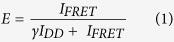



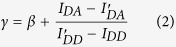



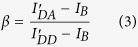


where β is given by the ratio of the donor emission fraction in the acceptor and donor channel, and γ is a relative brightness corrected factor given by


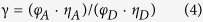


where *φ*_*A*_ and *φ*_*D*_ are the acceptor and donor quantum yields, respectively, and *η*_*A*_ and *η*_*D*_ are the detection efficiency for acceptor and donor fluorescence, respectively.

From the FRET efficiency, distances R can be obtained according to


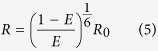


where R_0_ is the Förster radius. For Alexa 555 and Alexa 647, a distance of 51 Å was determined from the spectral overlap of the donor fluorescence and acceptor absorption.

### Setup for FRET-FCCS experiment

In the FCCS measurements, the experiment was performed on an inverted confocal microscope adapted for two-colour FCS and FRET systems. The sample was excited at a wavelength of 488 nm and a repetition frequency of 50 MHz and an intensity of 765 μW by a supercontinuum fiber laser (Fianium ultrafast fiber laser SC-400-4-PP) equipped with an acousto-optical tunable filter AOTF (Fianium AOTF PX-00027) and cleaned by a 512 nm shortpass filter (Semrock FF01-512/SP-25). The excitation light was reflected into a 60x immersion oil objective (Olympus PlanApo 60X; Cargille Type DF Immersion Oil) by a 532 nm dichroic mirror (Semrock FF535-SDi01). The fluorescent light was collected in an epifluorescence arrangement and is reflected by the dichroic mirror into the detector array. After a 50 μm pinhole (Thorlabs P50S) the signal is split into the donor and acceptor channels by a 649 nm dichroic mirror (Semrock FF649-Di01). The two channels are cleaned by bandpass filters at 550/88 nm for donor and 705/100 nm for acceptor (Chroma). Fluorescence emission was measured by the use of two high speed single-photon counting modules (Becker & Hickl HPM-100-40). The resulting photon count was recorded with a counter timer card (Magma ExpressCard/34) controlled by a TCSPC software suite (Becker & Hickl) to tag photon arrival times using the on-board 50 Mhz clock triggered with the laser excitation.

### FCCS references and measurements

We recorded single-photon auto-correlation (AC) and cross-correlation (CC) measurements on a confocal setup, which equipped with a combined FRET-FCCS module. Crosstalk between the donor and acceptor channels was determined by measuring the dyes separately. To designate the quality of the alignment and the associated parameters, each set of measurements was based on two reference measurements using Rhodamine 6G and double-labeled Poly-Proline with a size of 15 residues. Due to the wide emission spectrum of Rhodamine 6G, its fluorescence can be detected in both channels. Equalization of the two transit times through the focus is a tool to fine align the setup. Poly-Proline labeled with Alexa 488 and Alexa 647 as a rigid molecule provides significant FRET efficiencies to specify the sensibility of the FRET system used for Ykt6. Usable concentrations resulted in photon count rate between 1000 cps and 15000 cps and correlation amplitude of higher than 0.01. The solution was placed on the microscope slide and covered by a cover well (Item645501, GRACE BIO-LABS). The so determined system parameters are described in the supporting information.

### Global fitting and FCCS analysis

In order to determine characteristic constant parameters depending only on the instruments and labeling probes, this separate fitting model based on a non-linear least square algorithm was used^37^,^38^,^39^,^40^:


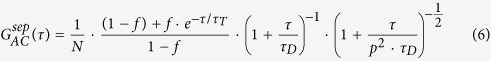



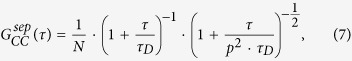


where AC indicates auto-correlation and CC cross-correlation, respectively. N is the average number of molecules in the focal region, f is the amplitude for the triplet component, and τ_T_ the triplet time constant. τ_D_ is the average transit time through the focal area and p = μ/s is the ratio of lateral (μ) to axial (s) dimension of the focus. Besides τ_D_, all parameter are constant and can be determined by reference measurements of Rhodamine 6G and double-labeled Poly-Proline.

The FCCS data of Ykt6 was analyzed by a global fitting model realized by the following non-linear least square multi-curve multi-parameter algorithm with global shared parameters which were showed in S2 Table^37^,^38^. The parameters *f*, τ_T_, and p were predefined by the separate fits by boundaries around the yielded values. The individual correlation functions are herein named as AC-d and AC-a for the ACs of donor and acceptor channels, respectively, as well as CC-ad and CC-da as their mutual CCs.


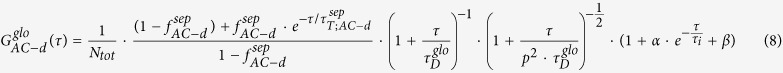



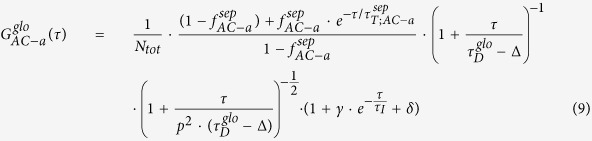



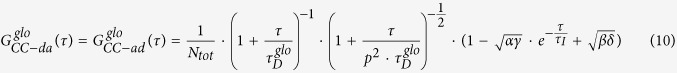


Here, the additional time dependency τ_I_ represents the time constant for the dynamics among the correlations and therefore the intramolecular dynamic. The parameters α and γ are scaling factors due to the correlation amplitudes; β and δ represent static contributions from FRET states that interchange on a much longer time scale than the transit time through the focus. The global uniform diffusion parameter τ_D_^glo^ is further corrected for AC-a by a fixed difference ∆.

### MD simulation parameters and system setup

We used the GROMACS 4.6.2^41^ software and CHARMM27 force field for protein^42^,^43^ and lipid^44^ in the MD simulations. The initial structure of apo Ykt6 was obtained from the crystal structure (PDB-ID: 3KYQ) by removing the bound DPC molecule. The missing residues at the C-terminal (residue 193–195) were built as described in our previous work^45^. The protein was solvated in a dodecahedron water box containing 20302 TIP3P water molecules^46^ to keep the protein 2.5 nm away from the edges of the box. NaCl was also added to the box at a concentration of 150 mM. The system was first energy minimized with a steepest descent algorithm by 1000 steps, followed by a 200-ps NPT simulation with position restraints on the heavy atoms and a 2-ns NPT simulation without any restraints. The temperature was kept at 300 K and the pressure was maintained at 1 bar using the weak coupling method. The cutoffs of VDW and short-range electrostatic interactions were both set to 1.2 nm, and long-range electrostatic interactions were calculated by the Particle-Mesh Ewald method^47^. All the bonds were constrained by the LINCS algorithm^48^ or by the SETTLE algorithm (for the bonds in water molecules only)^49^. The time step was set to 2 fs. All data analysis and structural visualization were done by VMD^50^.

### Metadynamics simulation

Metadynamics was firstly introduced by Parrinello’s group as a powerful method to explore the conformational space and determine the free energy surface of complex systems spanned on several collective variables (CVs) using a history-dependent potential term. The well-tempered scheme was adopted here, in which the height of added Gaussian potential is rescaled according to the history-dependent potential^51^. All the metadynamics simulations were performed by PLUMED plug-in version 1.3^52^ with GROMACS 4.6.2. The CV was defined as the distance between the Cα atoms of Cys66 and Glu175. The sampling region was restrained within 1.5~6.0 nm using restraining potentials, with energy constant of 500 kJ/mol, the rescaling factor of 1.0 nm, and the exponent determining the power law of 4. Three parallel simulations were carried out with different initial velocities and each lasted for 1500 ns. The system was maintained at 300 K. The initial Gaussian height was set to 2 kJ/mol, and the Gaussian width was set to 0.01 nm and the bias factor was 20. A new Gaussian potential was added every 2 ps.

## Additional Information

**How to cite this article**: Dai, Y. *et al*. Lipid Regulated Intramolecular Conformational Dynamics of SNARE-Protein Ykt6. *Sci. Rep.* **6**, 30282; doi: 10.1038/srep30282 (2016).

## Supplementary Material

Supplementary Information

## Figures and Tables

**Table 1:** Fitting parameters of Ykt6 at different Ykt6:DPC-ratios.

Ykt6:DPC	Correlation	N	f	τ_D_	p	τ_T_	rel. N	Δτ_D_
1:0	AC-d	7.287	0.282	208.477	6.900	3.813	1	0
	AC-a	45.001	0.331	191.845	6.900	8.827	6.176	−16.632
	CC-d	110.008		454.090	6.853		15.097	245.613
	CC-a	99.619		469.967	6.900		13.671	261.490
1:2	AC-d	8.066	0.284	209.990	6.900	3.224	1	0
	AC-a	34.240	0.343	175.049	6.850	5.247	4.245	−34.941
	CC-d	95.760		210.056	6.898		11.872	0.066
	CC-a	68.955		229.725	6.898		8.549	19.735

AC-d stands for autocorrelation donor; AC-a stands for autocorrelation acceptor; CC-d is the crosscorrelation donor; CC-a is the crosscorrelation acceptor. N is the average number of molecules in the focal region; f is the triplet value; τ_D_ is the average transit time through the focal area; p is the ratio of lateral to axial dimensions of the focal region; τ_T_ represents the triplet dynamics of the dyes; rel.N and Δ τ_D_ reflect relative molecule number and transit time.

**Table 2:** Global fitting parameters of FCCS data at different Ykt6:DPC ratios.

Fittingparameter	Ykt6:DPC = 1:0	Ykt6:DPC = 1:2
N	9.11 ± 0.53	8.87 ± 0.67
τ_D_ (μs)	220 ± 37	224 ± 7
τ_i_ (μs)	190 ± 55	15 ± 32
α	0.13 ± 0.04	0.04 ± 0.05
β	0.12 ± 0.05	0.07 ± 0.11
γ	1.01 × 10^−4^ ± 0.91 × 10^−4^	0.10 × 10^−2^ ± 0.88 × 10^−2^
δ	0.25 ± 0.06	0.15 ± 0.06

N is the average number of molecules in the focal region; τ_D_ is the average transit time through the focal area; τ_i_ represents the time constant for the dynamic among the correlations which is the intramolecular dynamic; parameters α and γ are scaling factors due to the correlation amplitudes; β and δ represent static contributions from FRET states. Compare the τ_i_ value between different ration of DPC, it shows that when the ratio increases to 1:2, the value of τ_i_ is in the range of the error bound of apo system.
