# Supplementary material for: Lipid Regulated Intramolecular Conformational Dynamics of SNARE-Protein Ykt6
Source: Sci Rep. 2016 Aug 5;6:30282. doi: 10.1038/srep30282 (PMC4974504; doi:10.1038/srep30282)
Supplement: Supplementary Information [file srep30282-s1.pdf]

Supplementary Information for:

Lipid Regulated Intramolecular Conformational Dynamics of SNARE-Protein Ykt6

*Yawei Dai[1, 2], Markus Seeger[3], Jingwei Weng[4], Song Song[1, 2], Wenning Wang[4], Yan-Wen Tan[1, 2, *]*

[1]*Department of Physics, Fudan University, 200433, Shanghai, P.R. China;*

[2]*State Key Laboratory of Surface Physics, Fudan University, 200433, Shanghai, P.R. China*

[3]*Institute of Biophysics, Goethe-University Frankfurt Max-von-Laue-Str. 1, 60438, Frankfurt/Main, Germany*

[4]*Shanghai Key Laboratory of Molecular Catalysis and Innovative Materials, Department of Chemistry and Institutes of Biomedical Sciences, Fudan University, 220 Handan Rd., 200433, Shanghai, P.R. China;*

[*]*Correspondence should be addressed to: ywtan@fudan.edu.cn.*

Note 1: Design of rYkt6ΔC FRET construct

Single molecule FRET (smFRET) is a direct and powerful technique for demonstrating the conformational changes between the longin domain and the SNARE core of rYkt6ΔC. Nevertheless, for smFRET measurements to perform well, the distances to be determined have to fall around the *Förster* radii R_0 of a chosen FRET pair since smFRET measurements are most sensitive there. The two labeling sites on the molecule, rYkt6ΔC, has to be chosen such that the distances between them will optimally report any conformational changes between the longin domain and the SNARE core.

There is a native Cysteine residue, C66, in the sequence of Ykt6 from *Rattus norvegicus* besides the CCAIM terminal. To minimize the perturbation of the original construct, we reserved this C66 in the longin domain as one of the FRET labeling sites. We then narrowed down our labeling site selection to the central part of the core domain. To choose the proper site, we fetched the longin domain sequences from Pfam and UniProt, ran multiple sequence alignment with ClustalWS, and calculated the conservation scores for each residue on rYkt6. Residues with low conservation scores was chosen as potential labeling sites. We have chosen E175 as our labeling residue on rYkt6ΔC. Since the only crystal structure of rYkt6 available (PDB-ID: 3KYQ) is in the closed form bound with DPC, we used MD simulation result to predict an open structure. Full length of rYkt6ΔC has 193 amino acids (Figure S1a), and the yielded distances between predicted label sites (C66 and E175C) are in the range of 20 Å and 62 Å for the closed and open conformations respectively. In this work we have chosen two different FRET pairs, Alexa 555/647 and Alexa 488/647, for smFRET and FCCS experiments, respectively. The *Förster* radii R_0 for these two pairs are 51 Å and 56 Å, which is suitable for reporting conformational changes between 20 Å and 62 Å. As a remark, previous work *Hasegawa et al. 2004* found that targeting function of Ykt6 is regulated by its longin domain (sequence: 1-137) and normal Ykt6 requires a soluble intermediate. In our earlier attempts, we found that the native cysteine C66 is a possible site related to the solubility of Ykt6. The final construct Ykt6/E175C keeps the soluble features as the wild-type one. Although the native C66 labeling site is not fully exposed to solution in the structure with PDB id 3KYQ, the construct rYkt6 E175C can be readily doubly labeled which is observed in single-molecule experiment. (see Figure S2).

Note 2: Structure diversity analyzed by MD simulations at a longer distances range

As the Cys66-Glu175 Cα distance falls within longer distance range, i.e. 3.2-3.9 nm, more prominent structural variability of the SNARE core was observed while the longin domain retains its structure. The conformations were divided into 49 clusters with a cutoff of 1.1 nm (Figure S7). The SNARE core becomes more stretched and less compact. The positions of the Glu175 Cα are obviously distributed in a larger space than that of the 'closed' state, ranging around the αA helix, the βC-βD turn, the αD helix and the βD-βE turn (Figure S8(a), (b)). In spite of the more extended conformations in the 'open' state, two clusters were still found with the longin-SNARE BSA of more than 12 nm2. The 1st cluster adopts a similar αG orientation as the 3rd and 5-8th clusters of the 'closed' state with the helix lying parallel with αA (Figure S8(c)). The 2nd cluster shows an antiparallel orientation between the αG and αA helices, reminiscent of the 9th cluster of the 'closed' state (Figure S8(d)). The N-terminus of αG helix is uncoiled in these two clusters, resulting in the longer distance between Glu175 and Cys66. We also inspect the centre structures with the smallest BSA (Figure S8(e-j)). In these structures, the αG helix is more than 0.9 nm away from the αA helix and has little contact with the longin domain. Nevertheless, the αG helix tends to pack with SNARE core itself in the last six clusters, placing its C-terminus in the vicinity of the αE helix. The backbone hydrogen bond interactions between βC and βF strands could be disrupted in the open state as in the last two clusters (Figure S8(i-j)). The disruption further reduces the BSA, providing extra conformational flexibility to the SNARE core. Though the open state is more disordered, the αG-αA packing is still observed, indicating the stability of the secondary structures.

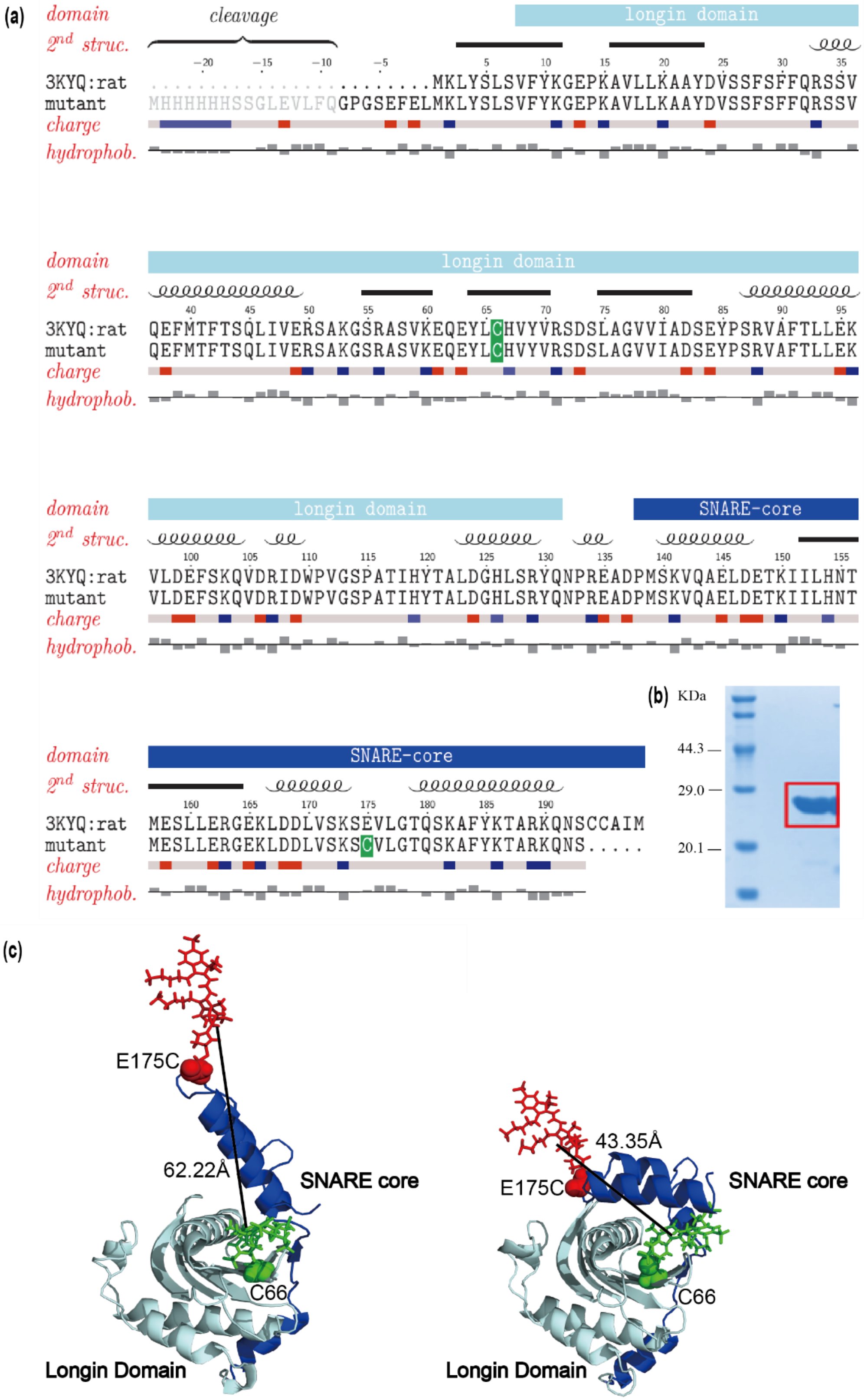
(a)
domain
cleavage
longin domain
2nd struc.
3KYQ:rat ........................MKLYSLSVFYKGEPKAVLLKAAYDVSSFSFFQRSSV
mutant MHHHHHHSSGLEVLFQGPGSEFELMKLYSLSVFYKGEPKAVLLKAAYDVSSFSFFQRSSV
charge
hydrophob.
domain
longin domain
2nd struc.
3KYQ:rat QEFMTFTSQLIVERSAKGSRASVKEQEYLCHVYVRSDSLAGVVIADSEYPSRVAFTLLEK
mutant QEFMTFTSQLIVERSAKGSRASVKEQEYLCHVYVRSDSLAGVVIADSEYPSRVAFTLLEK
charge
hydrophob.
domain
longin domain
SNARE-core
2nd struc.
3KYQ:rat VLDEFSKQVDRIDWPVGSPATIHYTALDGHLSRYQNPREADPMSKVQAELDETKIILHNT
mutant VLDEFSKQVDRIDWPVGSPATIHYTALDGHLSRYQNPREADPMSKVQAELDETKIILHNT
charge
hydrophob.
domain
SNARE-core
2nd struc.
3KYQ:rat MESLLERGEKLDDLVSKSEVLGTQSKAFYKTARKQNSCCAIM
mutant MESLLERGEKLDDLVSKSCVLGTQSKAFYKTARKQNS.....
charge
hydrophob.
(b)
KDa
44.3
29.0
20.1
(c)
E175C
62.22Å
SNARE core
C66
Longin Domain
43.35Å
E175C
SNARE core
C66
Longin Domain

Figure S1. Sequence, purification, and labeling strategy of our FRET construct rYkt6ΔC. (a) Amino acid sequence of rYkt6ΔC and the modified FRET-construct; The chosen labeling sites are C66 and E175C (highlighted in green and red). (b) SDS-PAGE gel showing the purified rYkt6ΔC protein used in our studies. The red box points out the molecular weight of rYkt6ΔC at 24.6 KDa. (c) Two structures predicts by our Molecular Dynamics (MD) simulations showing the structures of doubly labeled Ykt6 in open (left) and closed (right) states. Both structures are simulated based on PDB structure 3KYQ, which is closer to the closed form. Structures of dyes are simulated with assumed structures of Alexa 555 (green) and Alexa 647 (red).

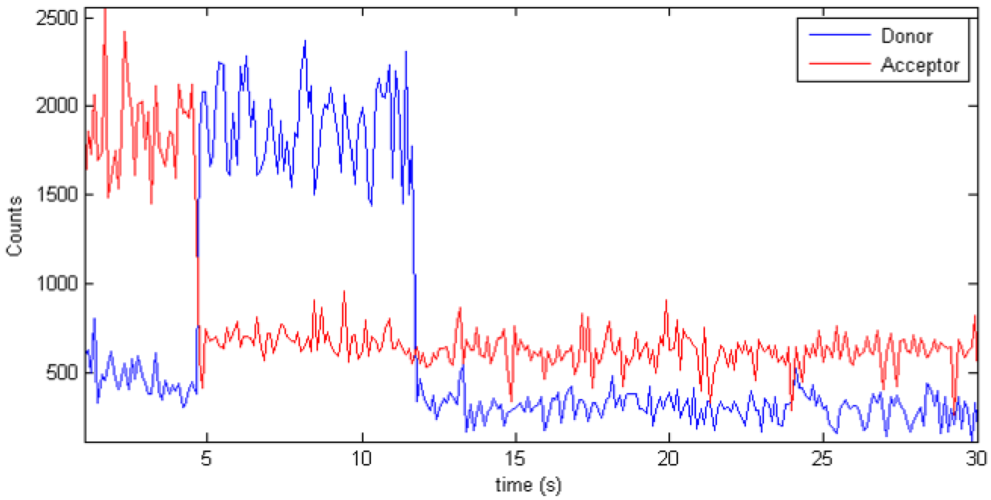

Figure S2. Single intensity vs. time trajectory of rYkt6ΔC. The rYkt6ΔC molecule is selected by the Co-localization method. The intensity vs. time trajectory of the chosen molecule is shown in Fig. S2. We impose a strict screening procedure to make sure each molecule taken into our data pool are labeled with one FRET pair (Alexa 555/647) as shown in Fig. S2. The blue curve stands for donor channel and the red curve stands for acceptor channel.

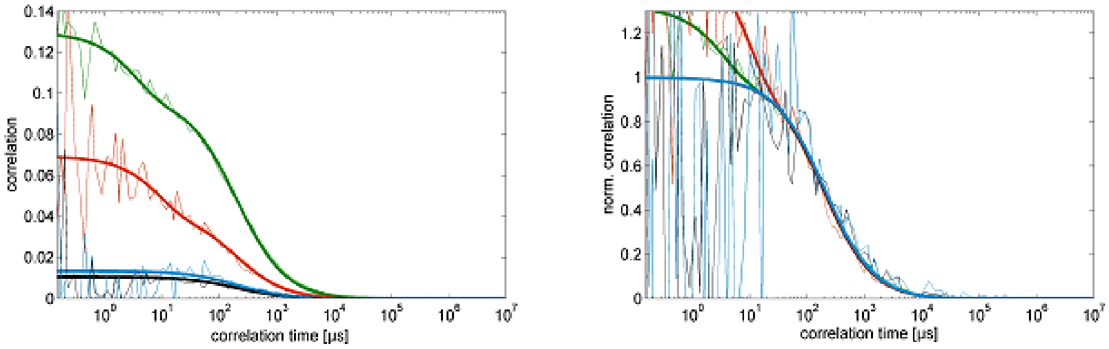

(a) raw (b) normalized

Figure S3. Correlations of double-labeled Poly-Proline (green: AC-d, red: AC-a, black: CC- da, blue: CC-ad). From Fig. S3(a) we found that all ACs (Autocorrelation) and CCs (Crosscorrelation) are obviously measurable. Regarding the correlation amplitude, the measurements confirms the expectations of a high amplitude of the AC-d (Autocorrelation Donor), a lower amplitude of acceptor and even lower amplitudes for the CCs. Furthermore the similarity of the CC shows the symmetry of the CCs. Fig. S3(b) shows that the normalized correlations owning the same correlation decay. This confirms that there is no time dependent difference between the four correlations. Since Poly-Proline$_{15}$ can be seen as a rigid molecule without intramolecular conformational dynamics, these measurements are consistent with theoretical expectations. Regarding the yielded Poly-Proline fitting results shown in tab. S1 , one can use the p-value marking the ratio of lateral to axial dimensions of the focal region for further measurements. The threshold of this parameter were set to 6.85 as the lower and 6.9 as the upper boundary conditions.

correlation	N	f	τ_D	p	τ_T
AC-d	10.187	0.243	198.393	6.847	3.480
AC-a	23.519	0.387	190.011	6.904	9.086
CC-da	94.974		197.182	6.864	
CC-aa	74.980		202.461	6.877	

Table S1: Fitting parameters of Poly-Proline

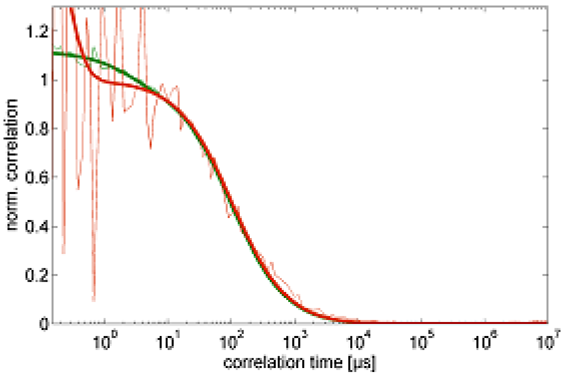

Figure S4. Normalized correlations of Rhodamine 6G with 1 nm and 5 nm (green: AC-d, red: AC-a). Rhodame 6G was used as a simple reference sample to calculate the dimension of the focal region. In Fig. S4, both correlation functions coincide with each other beyond 10 µs. From the fitting results one can calculate the dimensions of the focal region by using $\mu^2 = 4 \cdot \mathrm{D} \cdot \tau_D$ with µ as the lateral dimension of the focus, D as the specific diffusion coefficient of the sample and τ_D as the average transit time through the focus. This leads to the focal dimensions of 4.07µm x 0.59 µm.

correlation	N	f	τ_D	p	τ_T
AC-d	4.886	0.106	99.238	6.900	2.361
AC-a	27.236	0.661	104.901	6.900	0.157

Table S2. Fitting results of Rhodamine 6G

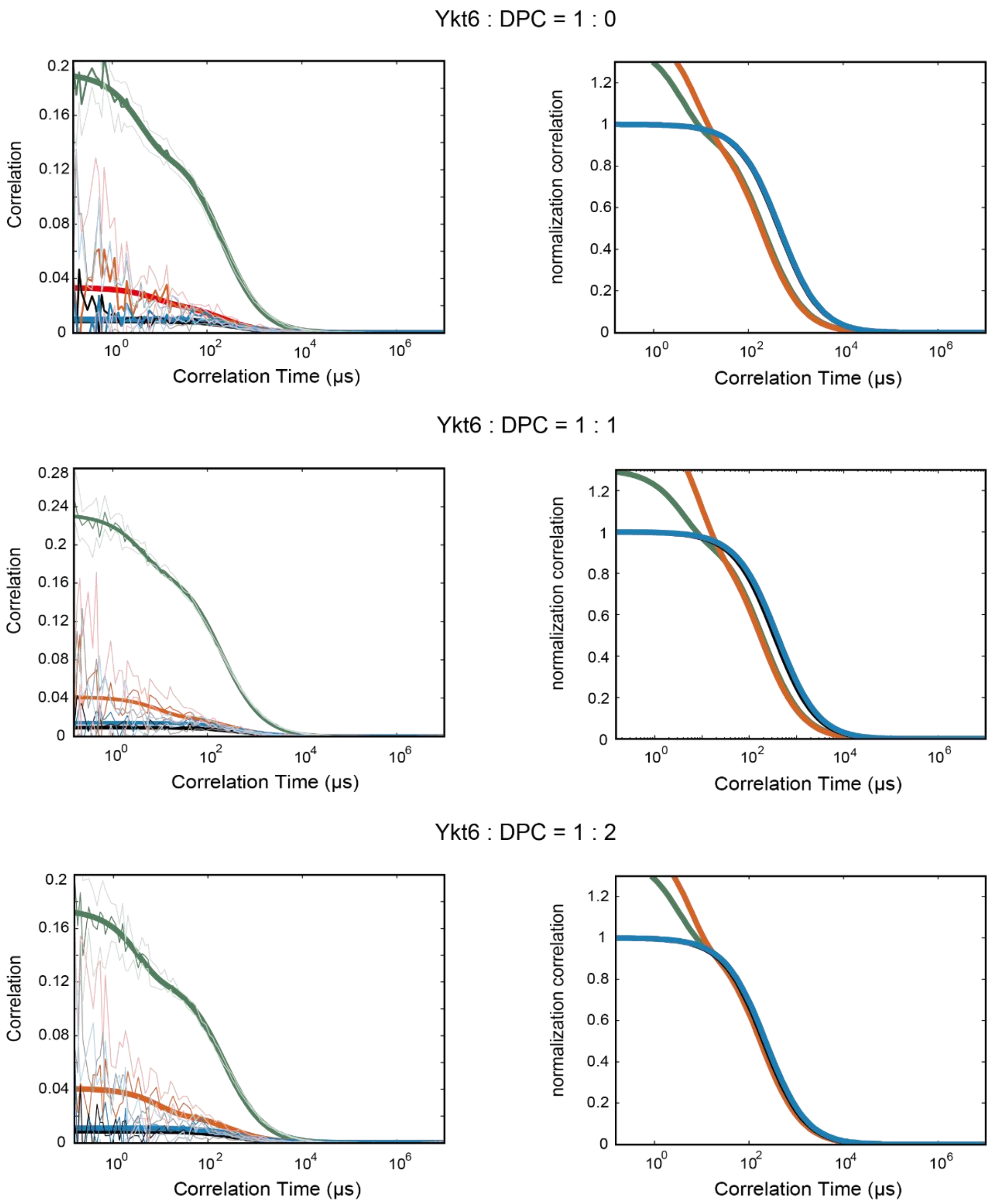

Figure S5. DPC concentration ratio gradient of double-labeled Ykt6 (green: AC-d, red: AC-a, black: CC- da, blue: CC-ad). Left side of Fig. S5 shows the raw data of correlations of double-labeled Ykt6. The normalized correlation data on the right side of Fig. S5 shows, that with increasing amount of DPC the time shift between both AC and the CC decreases and nearly vanishes at a concentration ratio of Ykt6 1:2 DPC. This DPC concentration ratio gradient presents that 1:2 ratio is the saturate concentration forces Ykt6 into a stable state.

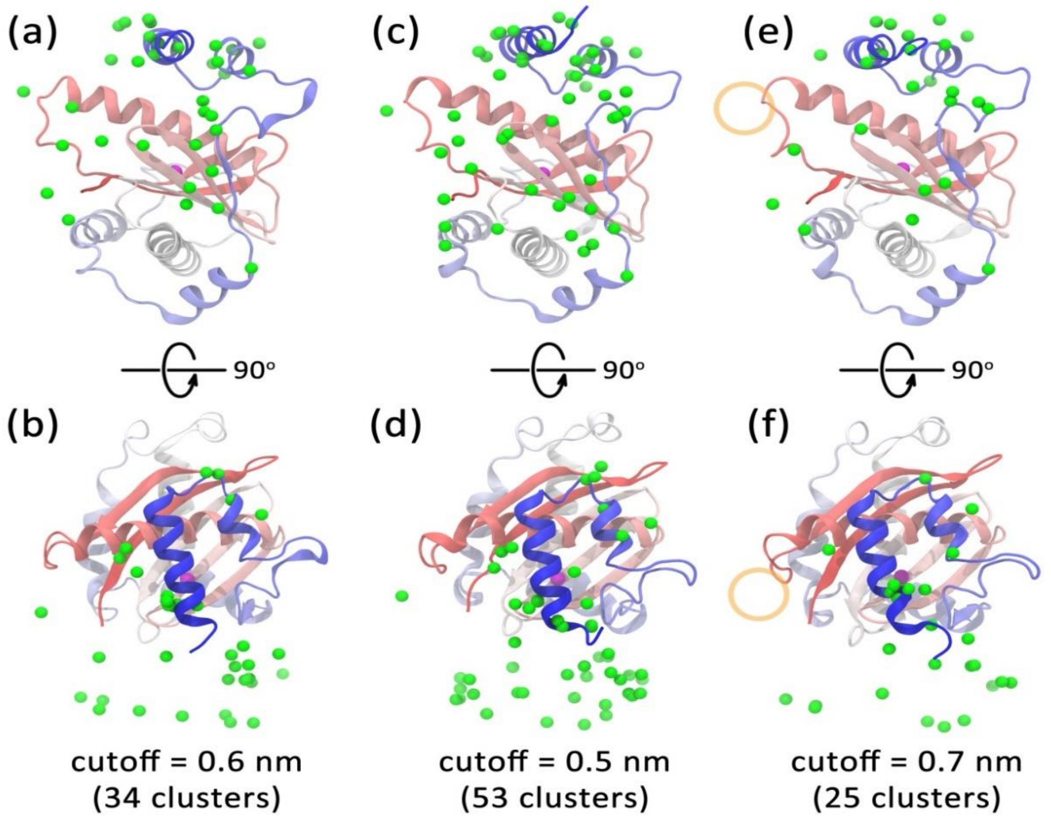

Figure S6. Cluster center structures of the snapshots with shorter Cys66-Glu175 distance in the metadynamics simulations using different cutoff values. The protein is represented by the cartoon mode and is coloured from red to blue. The Cα atoms of Glu175 in the cluster center structures are represented as green balls and the Cα atom of Cys66 is represented as magenta ball. Different cutoff values are used in the clustering analysis. Larger cutoff values lead to fewer clusters and when the cutoff equals 1.0 nm, a characteristic center structure is neglected as marked by the orange circle.

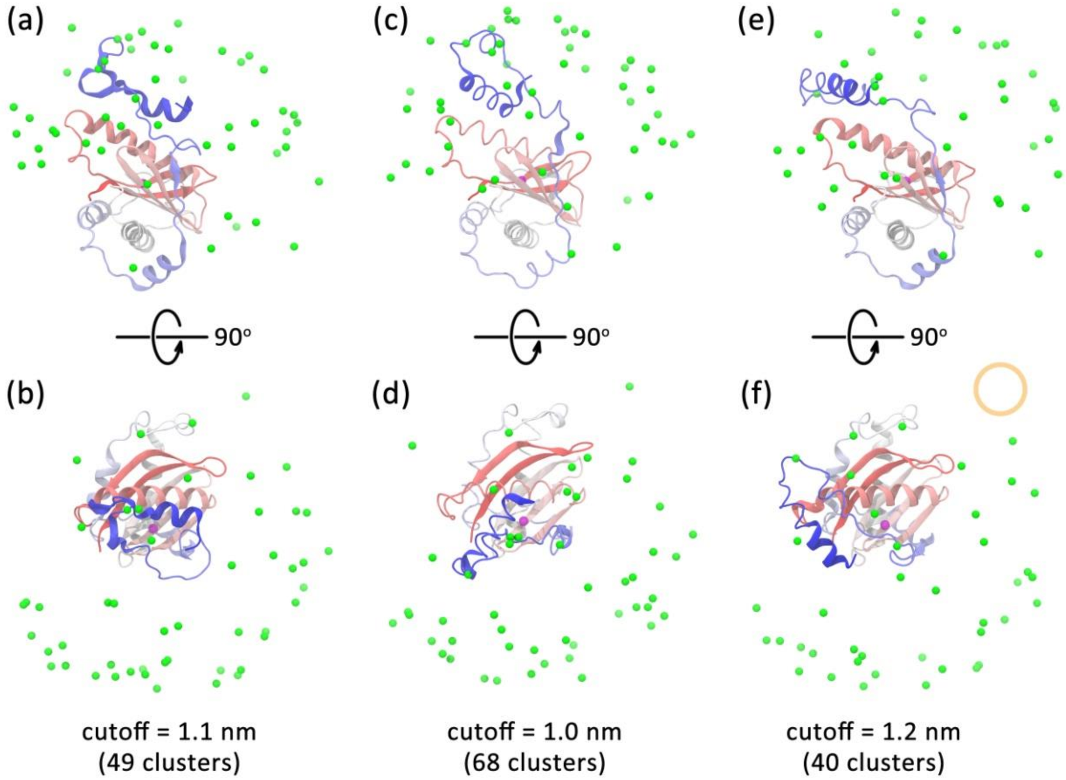

Figure S7. Cluster center structures of the snapshots with longer Cys66-Glu175 distance in the metadynamics simulations using different cutoff values. The protein is represented by the cartoon mode and is coloured from red to blue. The Cα atoms of Glu175 in the cluster center structures are represented as green balls and the Cα atom of Cys66 is represented as magenta ball. Different cutoff values are used in the clustering analysis. Larger cutoff values lead to fewer clusters and when the cutoff equals 1.0 nm, a characteristic center structure is neglected as marked by the orange circle.

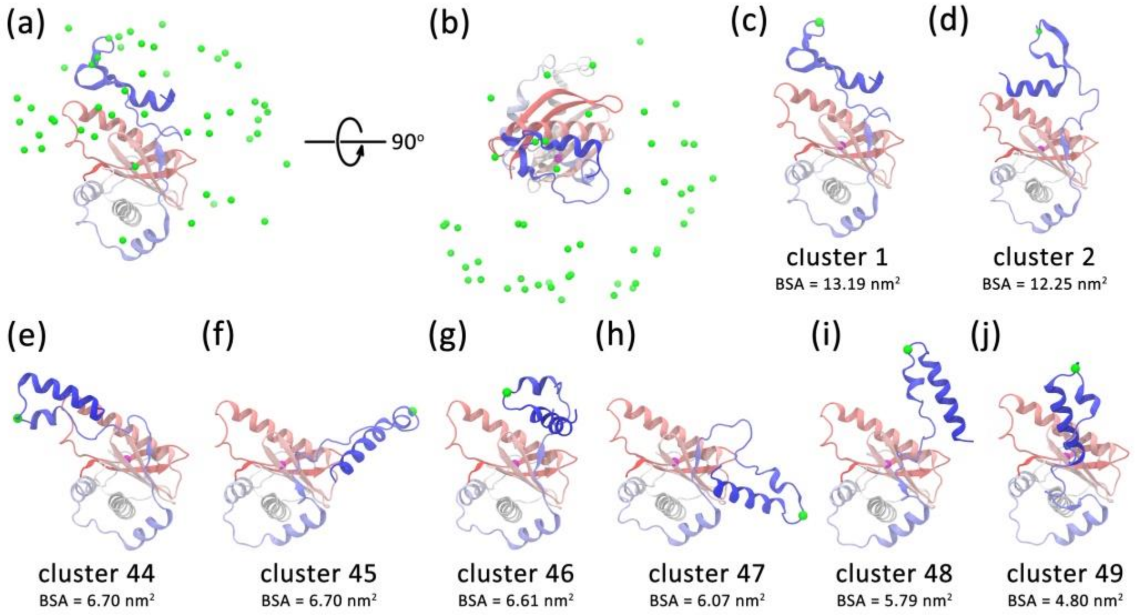

Figure S8. Representative structures with longer Cys66-Glu175 Cα distance from the MD simulation. Front view (a) and top view (b) of the cluster centre structures of the snapshots with the Cys66-Glu175 distance ranging from 3.2 to 3.9 nm in the metadynamics simulations using a cutoff of 0.6 nm. The protein is represented by the cartoon mode and is coloured from red to blue. The Cα atoms of Glu175 in the cluster centre structures are represented as green balls and the Cα atom of Cys66 is represented as magenta ball. (c)-(d) The centre structures with the BSA between longin domain and SNARE core of more than 12 nm^2. (e)-(j) The six centre structures with the smallest BSA.
